# Envisioning and Leading Organizational Transformation: One Organ Procurement Organization's Journey

**DOI:** 10.7759/cureus.879

**Published:** 2016-11-15

**Authors:** Jeffrey P Orlowski

**Affiliations:** 1 President & CEO, LifeShare Transplant Donor Services of Oklahoma

**Keywords:** opo, organ donors, organs transplanted, organizational transformation, opo transformation, donor potential, change, oklahoma, dcd, transplantation

## Abstract

Background: In 2012, one organ procurement organization (OPO) welcomed a new President and Chief Executive Officer (CEO). This OPO, LifeShare Transplant Donor Services of Oklahoma (LifeShare), had just celebrated its 25th anniversary in 2011. While LifeShare was well-established chronologically, growth in organ donors and organs transplanted from these donors had occurred at a much slower rate during the collaborative era and afterward (2003-2011) than the donor/transplant growth the United States (US), as a whole, had experienced.

While this performance had been stable, it was in the lower quartile of US OPOs on a per capita basis (organs transplanted per donor), and conversion rates were unremarkable. It was the sense of the OPO and donation service area (DSA) constituents that there was an opportunity for growth. It was under this premise that the new CEO was recruited in late 2011 and assumed leadership in February 2012.

Method: It important to note that the new CEO (the author) found LifeShare possessed numerous significant assets upon which to build. These included a strong core of committed and dedicated staff, a supportive Board, supportive transplant centers, and a strong state donor registry. Therefore, it was apparent that, while achieving the DSA's potential would require a transformation of the organization, the transformation did not necessarily require replacing core staff, often a common step undertaken by new chief executives.

Beginning in 2012, the CEO sought to transform both the culture and the operation of the organization by focusing on a short list of key strategies. Culturally, three primary initiatives were undertaken: leadership development, staff development, and establishing "organizational clarity". Operationally, the primary focus was identifying organ donor potential and then, based upon the opportunities for improvement, focusing on operational policies and practices. As LifeShare's team began to identify pockets of unrealized potential donors, recognized best practices were deployed to areas of opportunity, including responding to all vented referrals, implementation of dedicated family requestors, broadening of already-existing in-house coordinator programs, and aggressive expansion of the donors after cardiac death (DCD) program.

Results: From 2008 through 2011, the four years prior to the organization beginning its change journey, LifeShare recovered 344 organ donors from which 1,007 organs were transplanted in 48 months. During the first 48 months of the change journey (2012 through 2015), 498 organ donors (+44.8%) provided 1,536 organs transplanted (+52.5%). DCD donors increased from 22 to 91 (+413.4%) and brain death (BD) donors from 322 to 407 (+26.4%). While the rate of growth is slowing somewhat, the first eight months of 2016 continue to show a percentage growth over 2015 in double digits for both organ donors and organs transplanted.

Discussion: Clearly, our results have been transformed and continue to be transformed. A cultural foundation for both leadership and staff, combined with a single-minded focus on maximizing recovery of potential organ donors and maximizing transplantation of every potential organ, has allowed us to achieve exceptional growth rates on a scale that has resulted in more than 500 additional organs transplanted and lives saved over the last four years when compared to pre-change results.

## Introduction

After 25 years of organizational history, LifeShare Transplant Donor Services of Oklahoma (LifeShare) underwent a change of executive leadership with the recruitment and retention of a new President and Chief Executive Officer (CEO). The new CEO (the author) assumed his duties on February 1, 2012.

The CEO was recruited with the understanding that the OPO Board, the transplant centers within the donation service area (DSA), and much of the staff of the organization were eager to see the organization achieve new, higher levels of performance and that the organization needed to be transformed due to recent issues on a number of levels.

During the recruitment process, the new CEO had discussed at length with all parties that two fundamental pillars of change the CEO would focus on from day one would be (a) significantly changing the organizational culture and (b) identifying and pursuing the maximum donor potential in the state of Oklahoma. While national donation levels had increased in the collaborative era from 6,457 organ donors in 2003 to 8,126 organ donors in 2011 (+25.9%), donors in Oklahoma had increased only by 8.8% over the same time (91 in 2003 to 99 in 2011) [1]. Similarly, deceased donor organs transplanted nationally had increased from 18,659 in 2003 to 22,518 in 2011 (+20.7%) while organs transplanted from Oklahoma deceased organ donors increased only 12.0% (208 in 2003 to 233 in 2011) [2].

## Materials and methods

During the interview process and upon assuming my new duties, I identified that the organization was not devoid of assets and, in fact, a number of key elements were in place, though not necessarily taken advantage of. These elements provided a starting point and resources to begin transforming the organization.

Chief among these was a strong core of professionals at both leadership and staff levels. Given some of the turmoil and challenges of the transition from the previous CEO through an interim period, this staff had seen a lot of uncertainty over 18 months. It was a testimony to the long-term employees (and not so long-term employees) who persevered and kept the organization going that they were both capable of rising to the challenge and were committed to seeing the organization grow.

In addition to this core staff, two additional key assets were an OPO Board committed to working with me as the new CEO to build the best OPO possible and support from the DSA transplant centers (TCs) who were willing to work with me to overcome operational challenges experienced in prior years.

### Cultural change

Changing the culture of any organization is a major challenge for leadership. Changing the culture of an organization that has had 25-plus years of performance at a particular level (whether good, mediocre, or bad) with little change is harder. While there was a core staff who were committed to the organization, none of those staff had any experience outside of LifeShare and, thus, had no perspective or experience with anything other than the established organizational way.

To change the culture, three key initiatives were undertaken. These were to undertake a structured, facilitated development of the leadership team; to undertake development of the staff, including adding more staff, new positions, restructuring job roles and departments, and investing in significant staff training across the board; and working to establish a clear core purpose for everything our OPO does, an initiative that ultimately led to what is called our “organizational clarity”.

Leadership development began with all leadership team members reading Lencioni’s “The Five Dysfunctions of a Team” and beginning to work on self-assessing and self-identifying dysfunctional leadership practices [3]. The Studer Group was engaged to provide a one-year leadership development program in conjunction with two other OPOs beginning in the fall of 2012. This was followed by engaging the Table Group to work exclusively with the leadership team on organization-specific needs.

**Figure 1:**
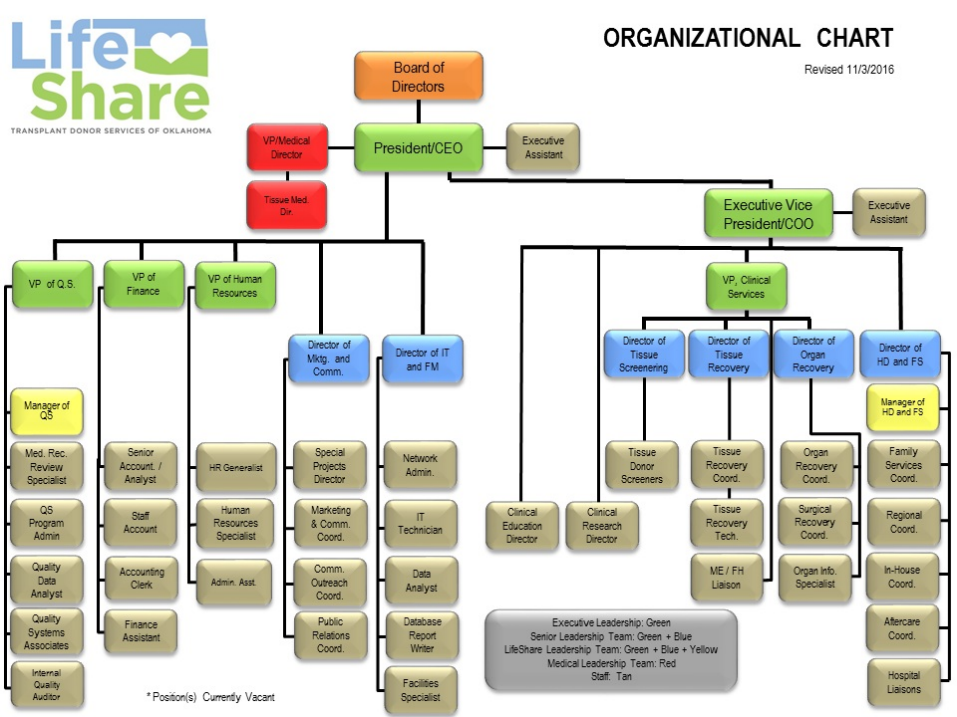
LifeShare Organizational Chart

Staff development began with reviewing both organizational structure and existing job descriptions. Growth in 2012 was fairly small as efforts to understand the potential and operational needs were undertaken. Late in 2012, additional positions began to be implemented and staff growth continues to this day. From a 2012 staff of 63 to a present day roster in excess of 110 staff (exclusive of contract and per diem employees), the organization has grown in every department and entire new teams (Family Services Coordinators, Regional Coordinators, and Surgical Recovery Coordinators, for example) have been added. The current organizational chart as of the time of this writing is depicted in Figure 1.

The growth of staff occurred both proactively and in reaction to growth. On the proactive side, positions that raised visibility and/or increased service were invested in. Examples include additional in-house coordinators, dedicated requestors or family services coordinators, and additional hospital development. These positions allowed our OPO to focus on expanding service to donor families and hospitals and to realize more donors by increasing authorization rates, expanding time and staff available for evaluation of extended and donors after cardiac death (DCD) donors, and expanding the LifeShare presence in previously underserved areas of the DSA. On the reactive side, as volume began to increase, positions added to meet the increase in demand included additional organ recovery coordinators, surgical recovery (perfusion) coordinators, and administrative support staff in accounting, quality, human resources, and donor family aftercare.

Staff development has also included a significant commitment to employee training. Our OPO now has a Clinical Training Director, has developed an internal training program called “LifeShare Academy”, and recently acquired an in-house clinical simulator. This simulator, a Laerdal SimMan® 3G (Laerdal Medical, Laerdal, Norway), is an advanced patient simulator that can display neurological symptoms as well as physiological symptoms and features. SimMan® 3G comes with a long list of features that will optimize simulation training scenarios, including automatic drug recognition, light sensitive pupils, bodily fluid excretion, and Wi-Fi portability. Acquired at a cost of over $100,000, SimMan® will allow us to conduct various advanced life support training programs, including advanced cardiac life support (ACLS) as well as other clinical training scenarios specifically programmed and tailored to common donor evaluation/ management challenges faced by our staff. In addition, the staff routinely attend a wide variety of external training programs and multiple programs, such as the bereavement-centered care training for family approach, which are brought in each year for a wide variety of staff roles.

**Figure 2:**
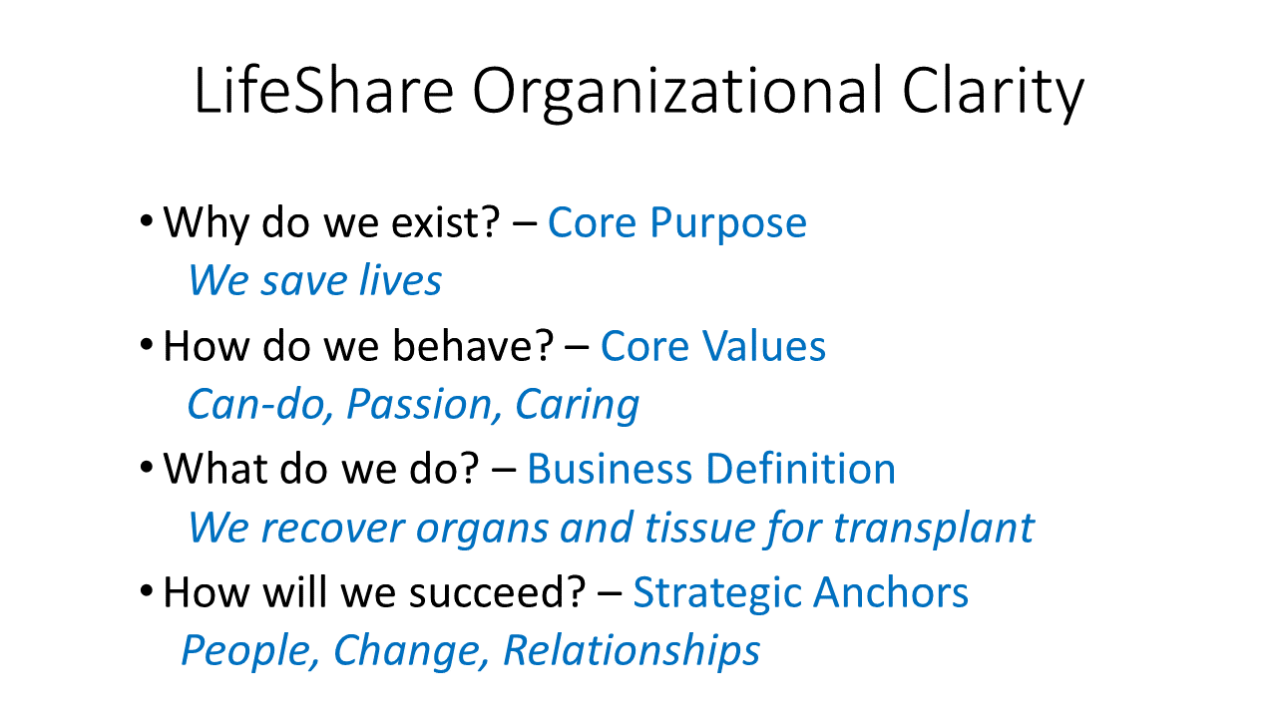
LifeShare Organizational Clarity

Finally, in the work done with the Table Group, leadership with input from the staff sought to answer four questions and in so doing established what is known at our company as “organizational clarity" (Figure 2). The questions describe what the organization’s purpose is, what its business is, how it behaves, and how it will succeed. All employees hear about the organizational clarity during recruitment, throughout orientation, routinely as teams face challenges and choices, and as a required component of every individual’s performance review.

Clearly, this level of staff growth, along with investments in staff training and leadership development, have come at additional expense, most of which falls into organizational overhead (indirect expense as well as general and administrative expenses). The total overhead cost has risen by 73% when the 2012 actual is compared to the 2015 actual. However, this increase in overhead has been spread over significantly more transplantable organs, resulting in the growth of acquisition charges at a much slower rate. In fact, comparing charges from January 2012 to 2015 year-end, renal acquisition charges have increased by 27%. Extra-renal acquisition charges have increased by 20% over the same time.  

Whether as in investment intended to increase activity or as a reaction to increased activity, the additional overhead expense has been largely absorbed by increased volume. Further, the author would argue that an annual increase in actual charges of roughly 5-6% in return for saving more than 500 additional lives over a four-year period is a quite reasonable investment in today's environment.

While cultural change is ongoing and, by self-definition (see “Organizational Clarity”, strategic anchor of change), will continue throughout our organizational journey, it is also clear that the organization is a far different organization culturally than it was prior to 2012. Today, LifeShare is a dynamic, growing organization that is focused on its core purpose…saving lives.

### Potential

Historically, deceased donor potential has been very difficult to define. While numerous attempts have been made to define the nation's donation potential and numerous hypotheses have been advanced, there is no clear agreement on how to define the number of potential donors and US estimates vary widely, from 13,000 to in excess of 30,000 [4-5]. While estimates vary, there is a general agreement that (a) there is an opportunity to increase donation nationally and (b) that much of the growth potential lies in donors that are “extended” in some way.

Beginning in 2012, our team sought to cast a wider net and to both track and hold ourselves accountable for maximizing the broadest donor pool we felt could reasonably be defined. We established a definition of potential that includes any death or imminent death our team believes has one or more transplantable organs and which is either (a) brain dead, regardless of age, (b) is 0-65 years of age and has a DCD potential, or (c) has a condition that precludes “eligibility” but we believe we can place an organ for transplant.

This definition was established in the summer of 2012. Our colleagues in Utah and Arizona generously shared an Excel-based tracking tool, one they had developed internally. The tool is not commercially available but interested parties may reach out to the author for more information. Beginning in 2013, our organization began tracking data in this tool and continue to do so to this day.

A key component of this process is a weekly “potential” meeting held to review vented referrals and deaths. Eligible and potential donors from referrals as well as those identified on death record review are tracked, evaluated, and categorized as “eligible”, “potential”, or "none". This meeting is attended by leadership and team members from organ recovery, hospital development and family services, quality systems, and executive leadership.

Data is trended by quarter and year, both for all of the OPO's hospitals in total and on an individual donor hospital basis. Reports are shared with donor resource teams and key hospital contacts at all donor hospitals. OPO leadership also uses data to target occurrences for follow-up (such as missed or late referrals, inappropriate approaches by hospital staff, etc.), to recognize improvement at donor hospitals, and to target resources. Examples of how resources have been targeted include:

- Expansion of In-House Coordinator program from three to seven full-time employees and from two to four hospitals. - Increased size of Hospital Development staff and targeted staff to key hospitals systems, like-hospitals, and geographically similar entities. - Increased organ staff to respond to all ventilated referrals; “call and we will come”. - Added mid-level management to provide hands-on leadership for staff in key roles of hospital development, family services, and quality systems, as well as additional executive leadership in clinical operations.

During the collaborative era, a goal of converting 75% of eligible donors was established, this goal has been met; yet, it is clear there is still an unmet need for transplantable organs. In evaluating potential, our organization has set a goal of maintaining a minimum 60% conversion of potential.

## Results

From January 1, 2013 through December 31, 2015, 675 deaths that met the “potential” definition were identified. Of these 404, were recovered donors or a potential conversion rate of 59.9%.

In 2015 alone, the potential review identified 292 potential donors in our DSA. Of these, 174 were converted to recovered donors, a conversion rate of 59.6%. Of these, 132 were brain dead donors of any age (75.9%) and 42 were DCD donors (24.1%).

Aggressive pursuit of these potential donors and aggressive efforts to place every organ by improving function pre-recovery through donor management, expanding the use of renal machine preservation, and, where necessary, expediting placement, along with a cultural change begun in 2012, has fueled a remarkable growth cycle. From 2008 through 2011, the four years prior to the organization beginning its change journey, our organization recovered 344 organ donors from which 1,007 organs were transplanted in 48 months.

**Figure 3:**
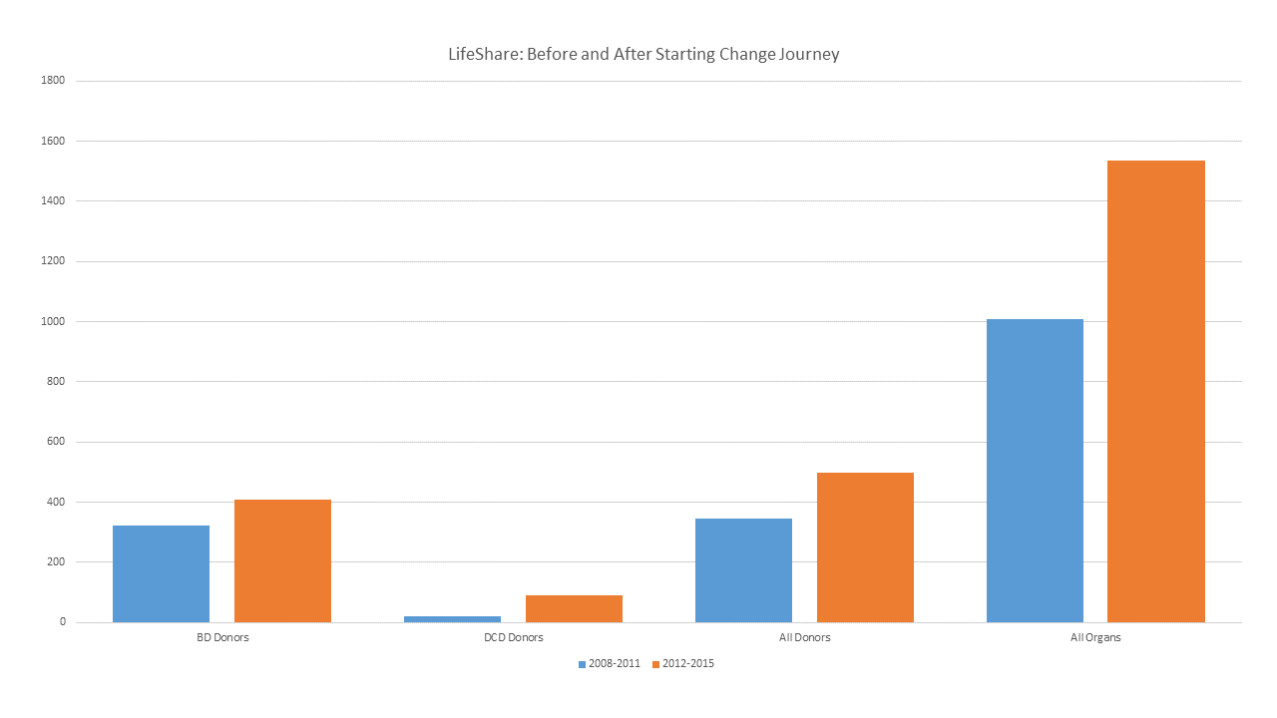
LifeShare Change Journey BD: brain death; DCD: donors after cardiac death

During the first 48 months of the change journey (2012 through 2015), 498 organ donors (+44.8%) provided 1,536 organs transplanted (+52.5%). DCD donors increased from 22 to 91 (+413.4%) and brain death (BD) donors from 322 to 407 (+26.4%) (Figure 3). While the rate of growth is slowing somewhat, the first eight months of 2016 continue to show a percentage growth over 2015 in double digits for both organ donors and organs transplanted.

## Discussion

Clearly, our OPO's results have been transformed and continue to be transformed. A cultural foundation for both leadership and staff, combined with a single-minded focus on maximizing the recovery of potential organ donors and maximizing transplantation of every potential organ, has allowed us to achieve exceptional growth. More than 500 additional organs have been transplanted over the last four years, resulting in more than 500 additional lives saved when compared to pre-change results.

It is important to note several important lessons learned. First, this transformation has come as a result of both proactive investment in the organization and its staff, as well as appropriate additional investment in reaction to increased activity. While it is the author's opinion that separating the impact of each form of investment is impossible, the overall effect has clearly produced significantly improved results.

It is likewise difficult-to-impossible to measure the individual impact of training versus increased staffing versus cultural change. Ultimately, the change journey that LifeShare has been on is a journey of complex change interweaving a number of simultaneous strategies. The intention of the author is not to relate the relative impact of each strategy but rather to paint a picture of what is possible when executive leadership targets strategic improvement on multiple fronts at the same time. Regardless of the relative impact, the author is quite confident that the strategic change package initiated in 2012, and which has evolved over the interim, has had the desired effect of achieving organizational transformation and thereby saving significantly more lives.

Of further interest is that, when the goal of converting 60% of potential was set, this projected to recovering 125-140 organ donors. However, as our staff and leadership have become more familiar with the potential definition and tool, and as the team has had success with recovering more extended and DCD donors, we have identified an increasingly larger “potential” than we at first believed was present. While we initially believed our potential to be around 200 in 2013 and 2014, we now believe potential in our DSA is in the 300 to 320 range. At a conversion rate of 60%, this would project to between 180 and 192 donors. In 2015, our team recovered 174 organ donors, which appears to justify the potential projected by this tool.

**Figure 4:**
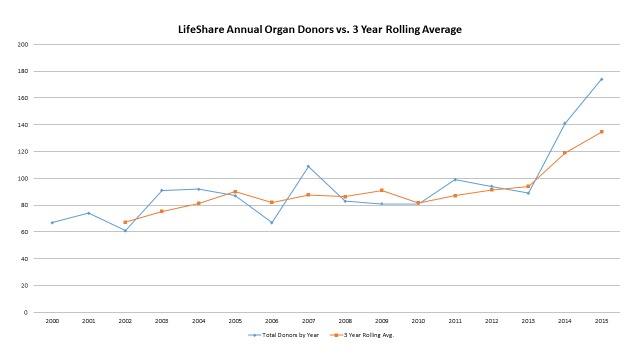
LifesShare Organ Donors by Year and 3-Year Rolling Average

**Figure 5:**
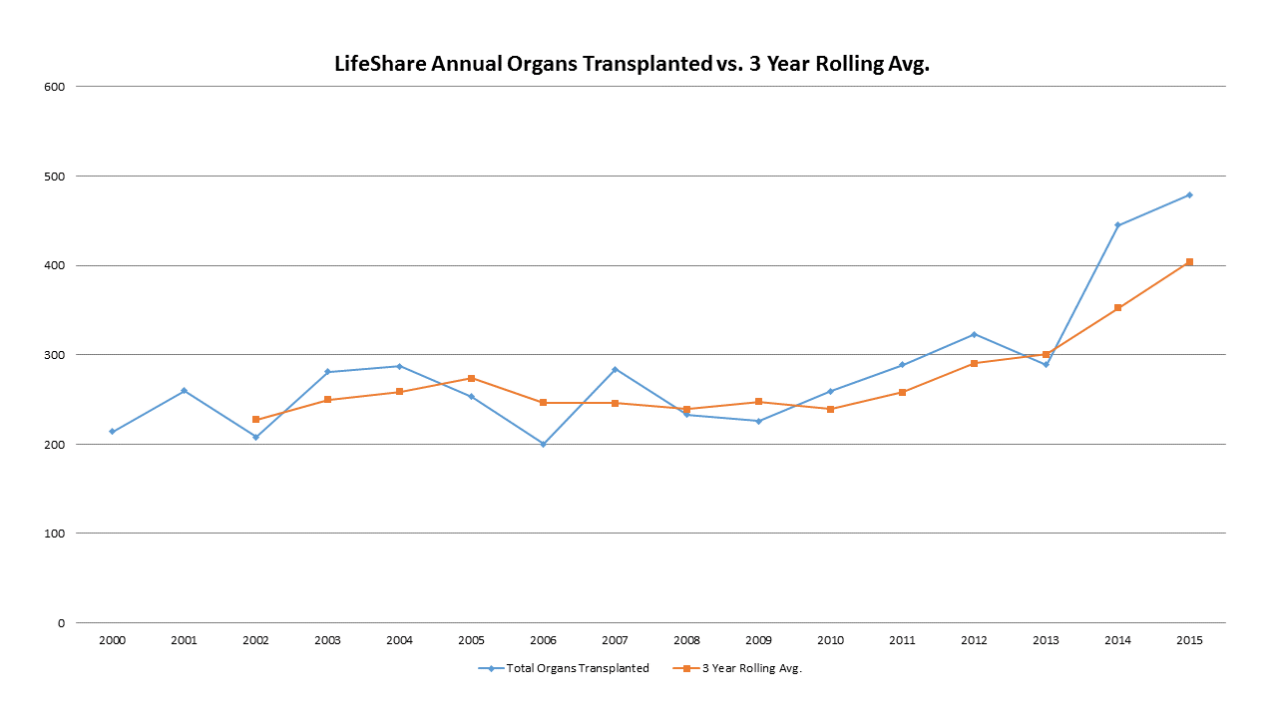
LifeShare Organs Transplanted and 3-Year Rolling Average

Worth noting is that, as the organization evolves, the areas of growth we are experiencing are also evolving. Early in our experience, growth was significant in both the brain dead and DCD populations. However, as recovery numbers have continued to rise year over year, an increasing percentage of the growth is occurring in DCD donors and extended donors (older donors or donors with significant co-morbidities likely to yield fewer organs per donor). The effect of this can be seen in the relative curves of organ donor growth (both year over year and a three-year rolling average) and the organs transplanted (Figures 4-5).

Comparing the orange curves in these two figures (the three-year rolling average), one sees that the rate of growth in donors is occurring at a slightly faster rate than the rate in organs transplanted. The three-year rolling average for organ donors increased by 48% from 2012 to 2015 while the three-year rolling average for organs transplanted increased 39%. This is reflective of the reality that, as the percentage of total donors that are either DCD or extended donors increase, the yield per donor will decline and inevitably, the growth rate of organs transplanted will be slower than the growth rate in donors. In the traditional, organs transplanted per donor (OTPD) world, a slower rate of growth in transplantable organs (equating to lower OTPD) is not necessarily a desirable OPO outcome. Traditionally, OPO yield has been defined by historical Centers for Medicare and Medicaid Services OPO performance measures focused on increasing OTPD. However, our organization's core purpose of “we save lives” sees every organ as valuable. Our results reinforce the importance of not only aggressively pursuing growth but also changing the OPO culture to focus on a simple, singular goal of increasing the number of organs transplanted in total, thereby maximizing the number of recipients who receive a lifesaving organ transplant.

## Conclusions

In summary, by implementing an organizational transformation based on cultural change and operationally focusing on maximizing every potential donor and every potential organ, our OPO has been able to dramatically increase donor recovery and organs transplanted. The increased cost of these initiatives has been largely offset by a significant increase in transplanted organs recovered. In total, comparing four-year periods pre-change and post-change, over 500 additional organs were transplanted from a donor population of 3.8 million. 

While the organization has been transformed from its pre-2012 state, there remains a lot of work to do. Hardwiring the practices and culture that have driven change is necessary. Expanding and improving organizational quality systems is a continuing focus as well. While proud of the progress made, the LifeShare team remains committed to continuing our organization’s change and improvement journey.
